# Synthesis and Molecular Structure of the 5-Methoxycarbonylpentyl α-Glycoside of the Upstream, Terminal Moiety of the *O*-Specific Polysaccharide of *Vibrio cholerae* O1, Serotype Inaba

**DOI:** 10.3390/molecules20022892

**Published:** 2015-02-11

**Authors:** Peng Xu, Edwin D. Stevens, Alfred D. French, Pavol Kováč

**Affiliations:** 1 NIDDK, LBC, National Institutes of Health, Bethesda, MD 20892-0815, USA; E-Mail: xup3@mail.nih.gov; 2 Department of Chemistry, Western Kentucky University, 1906 College Heights Blvd., Bowling Green, KY 42101-1709, USA; E-Mail: Edwin.Stevens@wku.edu; 3 Southern Regional Research Center, US Department of Agriculture, 1100 Robert E Lee Blvd, New Orleans, LA 70124, USA; E-Mail: Al.French@ars.usda.gov

**Keywords:** *Vibrio cholerae* O1, glycosylation, glycosidation, crystal structure

## Abstract

The trimethylsilyl trifluoromethanesulfonate (TMSOTf)-catalyzed reaction of methyl 6-hydroxyhexanoate with 3-*O*-benzyl-4-(2,4-di-*O*-acetyl-3-deoxy-l-glycero-tetronamido)-4,6-dideoxy-2-*O*-levulinoyl-α-d-mannopyranosyl trichloroacetimidate followed by a two-step deprotection (hydrogenolysis over Pd/C catalyst and Zemplén deacylation, to simultaneously remove the acetyl and levulinoyl groups) gave 5-(methoxycarbonyl)pentyl 4-(3-deoxy-l-*glycero*-tetronamido)-4,6-dideoxy-α-d-mannopyranoside. The structure of the latter, for which crystals were obtained in the analytically pure state for the first time, followed from its NMR and high-resolution mass spectra and was confirmed by X-ray crystallography. The molecule has two approximately linear components; a line through the aglycon intersects a line through the mannosyl and tetronylamido groups at 120°. The crystal packing separates the aglycon groups from the tetronylamido and mannosyl groups, with only C-H…O hydrogen bonding among the aglycon groups and N-H…O, O-H…O and C-H…O links among the tetronylamido and mannosyl groups. A carbonyl oxygen atom accepts the strongest O-H…O hydrogen bond and two strong C-H…O hydrogen bonds. The geometric properties were compared with those of related molecules.

## 1. Introduction

**Scheme 1:**
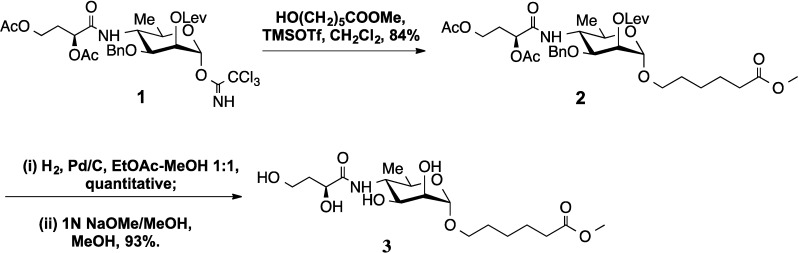
Synthetic route of Compound **3**.

O-specific polysaccharides (O-SP, O-antigens) are essential virulence factors and protective antigens of many pathogenic bacteria [1]. In Gram-negative bacteria, the same class of polysaccharides is responsible for the serological specificity of these pathogens. The O-SP of the two main strains of *Vibrio cholerae* O1, Inaba and Ogawa, consists of less [2] than 20 (**1**→**2**)-linked 4-amino-4,6-dideoxy-α-d-mannopyranosyl (perosaminyl) residues, the amino groups of which are acylated with 3-deoxy-l-*glycero*-tetronic acid. The two strains differ in that the terminal perosamine residue in the O-SP of the Ogawa strain is methylated at O-2 [3]. Following the pioneering work by Kenne *et al.* [4] on the synthesis of the methyl α-glycoside of the terminal, monosaccharide determinant of the O-SP of *Vibrio cholerae* O1, serotype Inaba, we have reported [5] an improved synthesis and the crystalline nature of the same compound. The presence of the methoxycarbonyl group in the title, spacer-equipped Compound **3** described here makes it amenable to conversions to an array of derivatives suitable for conjugation to proteins through different chemical processes. Thus, it will be useful, within *Vibrio cholerae* O1 strains, for making tools for immunological/immunogenicity studies towards elucidating the molecular basis for serotype specificity, which often require glycoconjugates. We have synthesized analogous substances from related oligosaccharides and converted them to conjugates [6] within our work towards a conjugate vaccine for cholera. The crystal structure of the complex from murine Fab S-20-4 (from a protective anti-cholera Ab specific for the lipopolysaccharide antigen of the Ogawa serotype) with synthetic mono- and di-saccharide fragments of the Ogawa O-SP has already been described [7]. The crystal structure of **3**, whose synthesis (Scheme 1) and isolation in the crystalline state and full characterization is described here for the first time, will aid in the interpretation of data resulting from a similar study in the Inaba series.

## 2. Results and Discussion

### 2.1. Synthesis

The known [8] trichloroacetimidate **1** was used as a glycosyl donor to couple with methyl 6-hydroxyhexanoate [9] under trimethylsilyl trifluoromethanesulfonate (TMSOTf) catalysis. Only αglycoside **2** was formed. The yield of **2** reported here (84%) is comparable or higher than when **2** was synthesized using acetate (88%) [10] or thioglycoside (70% α, 20% β) [10] as the glycosyl donor. All analytical data (^1^H-, ^13^C-NMR and HRMS) for **2** agreed with those reported [10]. Two-step deprotection (**2→3**) was performed by hydrogenolysis (5% Pd/C) followed by deacylation. The product of debenzylation, obtained in a virtually theoretical yield, was subjected to Zemplén transesterification. It simultaneously effected the removal of the acetyl and 2-*O*-levulinoyl groups, to give Compound **3** in a 93% yield, after column chromatography. Slow crystallization from MeOH gave crystals suitable for structural analysis by X-ray crystallography.

### 2.2. Crystallography

**Figure 1:**
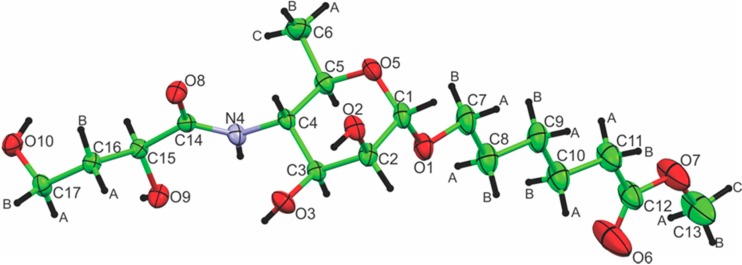
Thermal ellipsoid plot of **3**, at 50% probability for oxygen, nitrogen and carbon atoms. The number of the atoms is shown.

The details of the crystallographic determination are shown in Table 1. The molecule is shown in Figure 1 with atomic numbering for the heavy atoms, confirming the chemical and NMR analyses of the structure. The molecule has a pronounced bend; lines that connect C17 to C1 and C1 to C13 intersect with an angle of 120 °C due to the axial α-glycosidic bond and the *exo*-anomeric effect.

The molecule is amphiphilic, and the crystal is organized by both conventional O-H…O and N-H…O hydrogen bonds, as well as by van der Waals and C-H…O interactions. The hydrophilic portion of the molecule is formed by the O-2 and O-3 side of the perosaminyl residue, and liberal criteria for hydrogen bonds (as per the PLATON crystal analysis software) [11] yield seven conventional H-bonds. Six C-H…O bonds were also identified by PLATON, and two others were identified visually with lengths just slightly past the PLATON criterion. Such long bonds are feasible; in a recent atoms-in-molecules analysis of cellulose, a C-H…O bond as long as 2.83 Å had an electron density at its bond critical point of 0.004 e/au [12]. Support for stabilization from interactions having small O-H…O angles was found in studies of 1,2-dihydroxycyclohexane [13]. In those vacuum calculations for rotations of one of the hydroxyl groups, stabilizations of about 2 kcal/mol occurred despite an O-H…O angle of about 105° and a H…O length of 2.4 Å. This was also despite the absence of a confirmatory bond critical point. All proposed hydrogen bonds are shown in Table 2.

**Figure 2:**
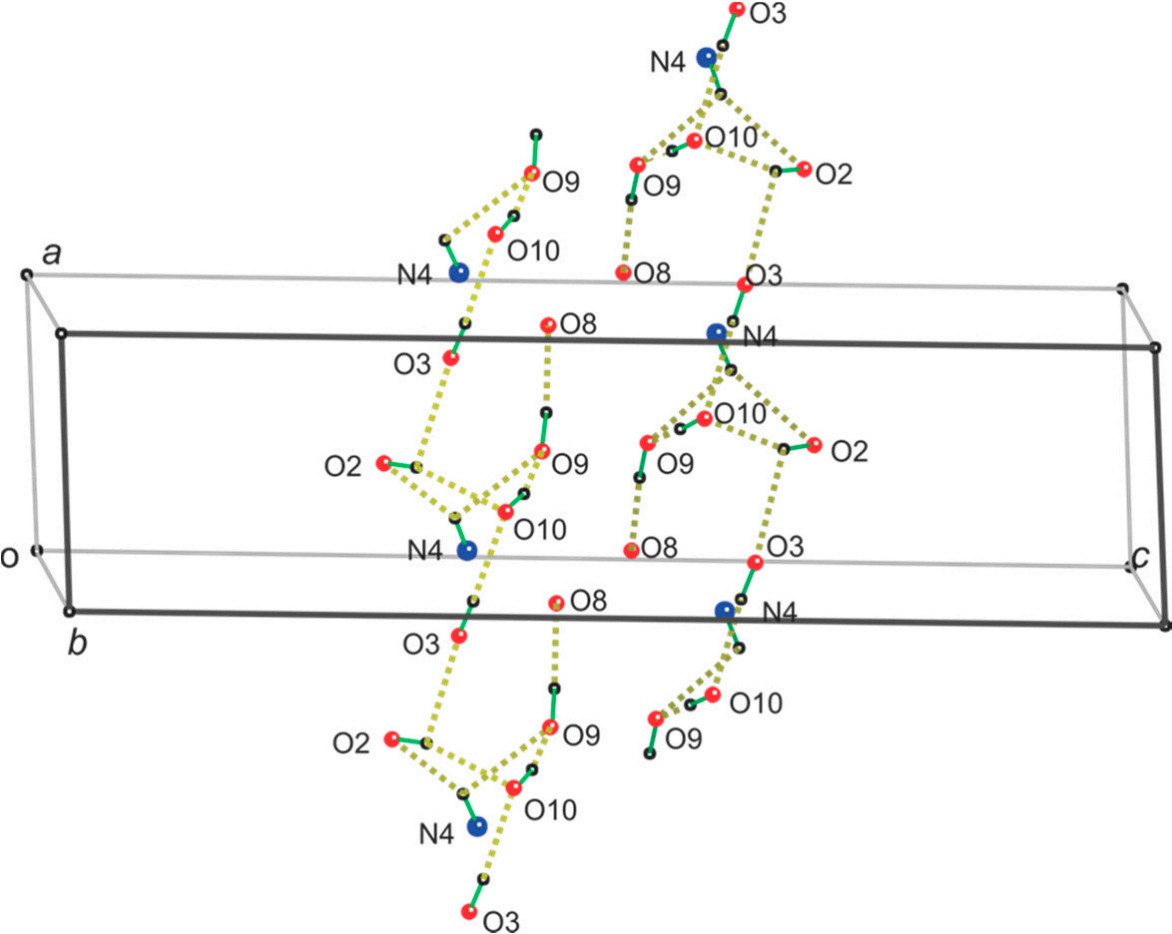
Two symmetry-related copies of the O-H…O and N-H…O hydrogen bonding network with the unit cell (One symmetry axis of this cell is coincident with the *b*-axis, and the other is parallel, but intersects the *a*–*c* plane at its center. The rings of the hydrogen bonds consist of N4-H, O2, O2-H, O10, O10-H and O9; infinite chains consist of O3, O3-H, O10, O2 and O2-H, which donates to O3, beginning the next repeat unit. The rings depend on N4 as a double donor and O9 as a double acceptor. In the infinite chains, O10 is a double acceptor. The strongest hydrogen bond is from O9-H to carbonyl oxygen atom O8. See Table 2 for the geometric values).

Figure 2 shows the conventional hydrogen bonding that consists of a ring and an infinite chain. All hydroxyl groups are both donors and acceptors. As shown in Table 2, the N4-H…O2 and O2-H…O3 links are of marginal quality (long H…O distances and small O-H…O angles) and were not reported by the ShelXL program used to refine the crystal structure. The double acceptor O10 permits the reversal of the nominal polarity of the hydrogen bonding (a fully cooperative network would have a “head-to-tail” donor-acceptor-donor-acceptor arrangement).

**Table 1:** Crystal data and structure refinement for **3**.

**Parameter**	C_17_H_31_NO_9_
Formula weight	393.43
Temperature	200(2) K
Crystal shape	needle
Color	colorless
Wavelength	0.71073 Å
Crystal system	Monoclinic
Space group	*P2_1_*
Unit cell	*a* = 5.66820(10) Å, *b* = 8.0033(2) Å, *c* = 22.1889(5) Å, β = 93.353(1)°, *V* = 1004.86(4) Å^3^, *Z* = 2
*d_calcd_*	1.300 Mg/m^3^
data collection	Bruker APEX-II CCD
Mo Kα	λ = 0.71073 Å (graphite monochromated)
Absorption coefficient	0.105 mm^−1^
*F*(000)	424
Crystal size	0.40 × 0.30 × 0.15 mm^3^
θ range	1.84 to 27.50°
Index ranges	−7 ≤ *h* ≤ 7, −10 ≤ *k* ≤ 10, −28 ≤ *l* ≤ 28
Reflections collected	16,355
Independent reflections	4597 [*R_int_* = 0.0158]
Completeness to θ = 27.50°	99.9%
Absorption correction	Semi-empirical from equivalents
Max. and min. transmission	0.9844 and 0.9593
Refinement method	SHELXL; Full-matrix least-squares on *F*^2^
Data/restraints/parameters	4597/1/270
Goodness-of-fit on *F*^2^	1.060
Final *R* ^a^ indices (I > 2σ(I))	R1 = 0.0301, wR2 = 0.0799
*R* ^a^ indices (all data)	R1 = 0.0318, wR2 = 0.0814
Absolute structure parameter	0.1(6)
Residual electron density (max, min)	0.238 and −0.178 e Å^−3^

^a^ R1 = Σ║*F_0_*│ − │*F_c_*║/Σ│*F_0_*│; wR2 = [(Σ*w*(*F_0_^2^* − *F_c_^2^*)^2^/Σ*w*(*F_0_^2^*)]^½^.

**Table 2:** Hydrogen bonds determined by PLATON criteria (hydrogen positions as determined) ^a^.

Bond D-H…A ^b^	Symmetry	d(D-H) (Å)	d(H…A) (Å)	d(D…O) (Å)	D-H…O (°)
O2-H…O3	Intra	0.82(2)	2.40(2)	2.7418(13)	106.2(18)
N4-H…O9	Intra	0.794(17)	2.266(16)	2.637(13)	109.3(13)
O2-H…O10	−x, y + ½, −z + 1	0.82(2)	2.10(2)	2.8692(14)	157(2)
O3-H…O10	−x + 1, y + ½, −z+1	0.78(2)	2.03(2)	2.8047(13)	176(2)
N4-H…O2	x + 1, y, z	0.794(17)	2.464(16)	3.1788(14)	150.4(15)
O9-H…O8	x + 1, y, z	0.81(2)	1.86(2)	2.6673(12)	174(3)
O10-H…O9	−x + 1, y−½, −z + 1	0.73(2)	2.05(2)	2.7323(15)	158(2)
C4-H…O8	Intra	1.00	2.43	2.8437(14)	104
C17-HA…O9	Intra	0.99	2.59	2.9858(16)	104
C2-H…O7	−x, ½ + y, −z	1.00	2.58	3.421(2)	142
C3-H…O2	1 + x, y, z	1.00	2.41	3.2689(13)	144
C4-H…O9	−1 + x, y, z	1.00	2.65	3.543	149
C16-HA…O8	−x, ½ + y, 1 − z	0.99	2.52	3.4912(15)	167
C16-HB…O3	−x, −½ + y, 1 − z	0.99	2.46	3.3401(15)	147
C9-HB…O6 ^c^	1 − x, −½ + y, −z	0.99	2.64	3.431	137

^a^ Numbers in parentheses refer to standard deviations for the last decimal place; ^b^ D-H…A represents the donor atom, the donated hydrogen and the acceptor atom, respectively; ^c^ this interaction was not detected by PLATON or Mercury [14] with the default criteria as such, although both found a close H9b…O6 short contact. Mercury’s criteria were adjusted to include carbon donors and a minimum D-H…O angle of 100° to prepare the drawings of Figure 3.

**Figure 3:**
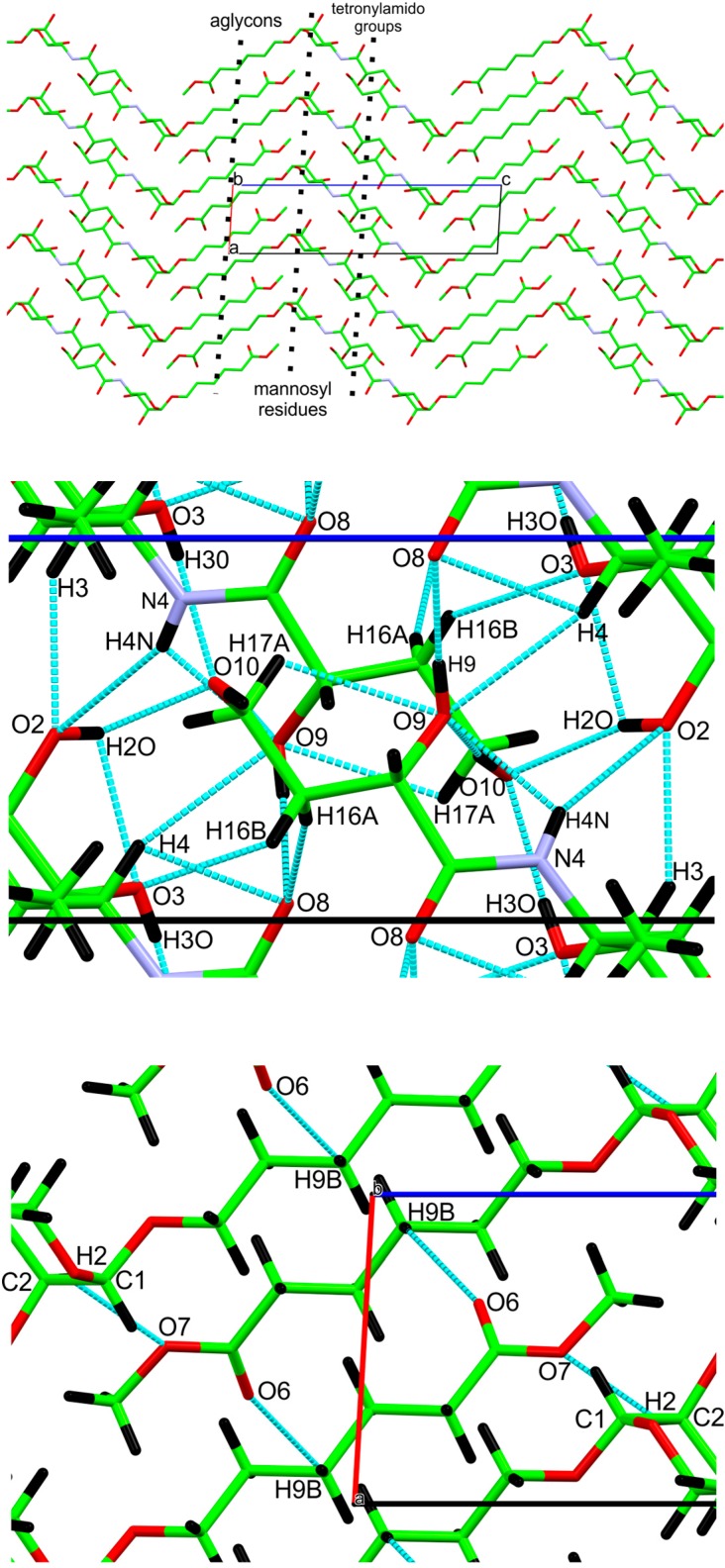
Crystal packing and hydrogen bonding, viewed along the *b*-axis of the unit cell. The upper drawing has the aglycon units along the *a*-axis, and the tetronylamido groups are located at the center of the unit cell. The mannosyl units are at about ¼ (and ¾) of the *c*-axis. The middle and lower figures show the details of the hydrogen bonding near the center and corner of the cell, respectively. The H2 atoms are hidden behind the C1-C2 bonds.

These hydrogen bonds are located near the two-fold screw axes that perpendicularly intersect the *a–c* plane. The C-H…O hydrogen bonds are more evenly distributed, as shown in Figure 3. The carbonyl oxygen O8 not only is the acceptor for the shortest O-H…O bond, but also is the acceptor for two short C-H…O hydrogen bonds, from H4 on the carbohydrate ring and from H16A on another tetronylamido residue (Table 2). The aglycon participates only in C-H…O bonds.

Cremer-Pople puckering parameters for **3** and three other molecules of this series of compounds are in Table 3. All are within the ranges observed for rings described as ^4^C_1_. Another measure of ring geometry is the distance across the ring, shown as the O1-N4 distance. The analogous O1-O4 distance for α-d-glucose determines (in a model-building sense) or is determined by (in an experimental sense) the location of substituents in the 1- and 4-positions (e.g., glucose residues in starch). The O1-N4 values are about 4.6 Å for this limited set of compounds, all near the upper end of the range (3.9 to 4.8 Å) observed for α-d-glucose [15].

**Table 3:** Ring geometry.

Structure	Puckering Q (Å)	Puckering Θ (°)	Puckering Φ (°)	O1-N4 (Å)
**3**	0.5615(13)	4.11(13)	249.2(17)	4.571
SUNFEM ^a^ [5]	0.577(5)	2.4(5)	207(10)	4.512
TEDJIV ^a^ [16]	0.550(3)	3.5(3)	166(4)	4.520
TEDJOB ^a^ [16]	0.527(10)	9.2(12)	259(7)	4.678

^a^ The six-letter codes are the “reference codes” used in the Cambridge Crystal Structure Database (CSD) [17]. The compounds are: SUNFEM, methyl 4,6-dideoxy-4-(3-deoxy-l-glycero-tetronamido)-α-d-mannopyranoanoside [5]; TEDJIV, methyl 4,6-dideoxy-4-(3-deoxy-l-glycero-tetronamido)-2-*O*-methyl-α-d-mannopyranoanoside monohydrate [16]; TEDJOB, methyl 4,6-dideoxy-2-*O*-methyl-4-trifluoroacetamido-α-d-mannopyranoanoside [16].

Geometric properties (Table 4) near the anomeric center (C1) are of continuing interest for carbohydrates, especially the C-O bond lengths and the *exo*-anomeric torsion angle, O5-C1-O1-C7. Values for the latter are nearly perfect embodiments of the *exo*-anomeric effect with a nominal value of 60°. The bond lengths of **3** are within one standard deviation of the mean values from a survey of the Cambridge Crystal Structure Database (CSD) that was restricted to structures with crystallographic R-values <0.05. With a crystallographic R factor of 0.07, bond lengths for TEDJOB (methyl 4,6-dideoxy-2-*O*-methyl-4-trifluoroacetamido-α-d-mannopyranoanoside) may not be accurate enough for this comparison; its O1-C7 length of 1.391 Å is shorter than all but one of the values in the CSD search; the second shortest of the 106 values was 1.410 Å.

**Table 4:** Properties around the anomeric center ^a^.

Structure	O5-C5 (Å)	O5-C1 (Å)	C1-O1 (Å)	O1-C7 (Å)	O5-C1-O1-C7 (°)
**3**	1.4425(14)	1.4116(18)	1.4085(15)	1.4322(18)	69.07(14)
SUNFEM [5]	1.437(6)	1.406(6)	1.392(6)	1.442(7)	61.6(5)
TEDJIV [16]	1.436(4)	1.405(4)	1.409(4)	1.441(5)	60.0(4)
TEDJOB [16]	1.421(13)	1.433(12)	1.420(13)	1.391(14)	67.2(11)
CSD Survey	1.434(9)	1.420(11)	1.400(10)	1.429(10)	66.8(54)

^a^ Numbers in parentheses for **3** indicate the standard deviation for the last digit, indicating the experimental uncertainty. Numbers in parentheses for the CSD survey indicate the standard deviation of the mean value for all 106 methylated pyranosides with the C5-O5-C1-O1 torsion angle restricted to 60° ± 30° (to assure an axial configuration of the C1-O1 bond).

Table 5 provides geometric details for the acetamido groups of the four related compounds, as well as a survey of the CSD. The first TEDJOB C-C value is very short, probably because of the disorder of the attached fluorines and high R factor. In **3**, the length of the carbonyl carbon to nitrogen bond (1.3309(15) Å) is much shorter than the nominally similar N-C4 bond (1.4648(14) Å). This difference is in excellent agreement with values from a search of the CSD. The endocyclic C4-C3 and C4-C5 bond lengths are normal, apparently not affected by the presence of the adjacent nitrogen.

**Table 5:** Structure of the tailing acetamido group.

Structure	C15-C14 (Å)	C14=O8 (Å)	C14-N4 (Å)	N4-C4 (Å)	C4-C3 (Å)	C4-C5 (Å)
**3**	1.5323(15)	1.2357(14)	1.3309(15)	1.4648(14)	1.5268(17)	1.5307(16)
SUNFEM [5] ^a^	1.525(7)	1.236(8)	1.326(7)	1.455(7)	1.521(6)	1.536(7)
TEDJIV [16]	1.527(4)	1.227(4)	1.325(4)	1.459(4)	1.527(4)	1.516(4)
TEDJOB [16]	1.40(2)	1.234(18)	1.316(18)	1.451(13)	1.515(14)	1.530(13)
CSD Search	1.521(17)	1.231(10)	1.336(12)	1.458(9)	–	–

^a^ The compounds of the SUNFEM, TEDJIV and TEDJOB crystal structures are in the footnote for Table 3.

Both the nitrogen and the adjacent carbonyl carbon atom have nominal *sp*^2^ hybridization that places them and their attached atoms in a common plane. The means of the absolute deviations from this plane are shown in Table 6. As reported in [5], there is a small deviation for SUNFEM, but as shown in Table 6, there is less deviation for **3**. There is even less deviation for the other structures or the mean deviations from the search of 4,086 structures in the CSD. Finally, the six atoms of the three related rings were fit to the ring in **3**, and the rings had a high degree of similarity, despite the variations in puckering and bond lengths.

**Table 6:** Miscellaneous.

Structure	Mean Deviation from C4-N4-C14-O8 Plane (Å)	RMS Deviation of Rings with 3 (Å)
**3**	0.025	–
SUNFEM [5]	0.035	0.0208
TEDJIV [16]	0.009	0.0258
TEDJOB [16]	0.007	0.0295
CSD Search	0.014(14)	–

## 3. Experimental Section

### General Information

Optical rotation was measured at ambient temperature with a digital Jasco automatic polarimeter, Model P-2000 (Easton, MD, USA). The melting point was measured on a Kofler hot stage. All reactions were monitored by thin-layer chromatography (TLC) on silica gel 60-coated glass slides. Column chromatography was performed by elution from prepacked columns of silica gel (Varian, Inc., Palo Alto, CA, USA) with the Isolera Flash Chromatograph (Biotage) connected to the external Evaporative Light Scattering Detector, Model 380-LC (Varian, Inc.). Nuclear Magnetic Resonance (NMR) spectra were measured at 600 MHz for ^1^H and 150 MHz for ^13^C, with Bruker Avance spectrometers (Billerica, MA, USA). Solvent peaks were used as the internal reference relative to tetramethylsilane (0 ppm). Assignments of NMR signals were made by homonuclear and heteronuclear two-dimensional correlation spectroscopy, run with the software supplied with the spectrometers. When reporting assignments of NMR signals, nuclei associated with the Tetronic side chain are denoted with a prime, and those associated with the spacer are denoted with a double prime. Liquid chromatography-electron spray-ionization mass spectrometry (ESI-MS) was performed with a Hewlett-Packard 1100 MSD spectrometer (Palo Alto, CA, USA).

Single crystal X-ray diffraction intensities were collected using a Bruker Kappa APEX II 4K CCD (Madison, WI, USA) 4-circle automated diffractometer and MoKα radiation. During data collection, the sample was cooled to 200(2) K using a stream of cold N_2_ gas generated with an Oxford Cryosystems 700 low-temperature system. The crystal structure was solved [18] using SHELXS-97 and refined using SHELXL-97 [19]. The absolute configuration of the structure was established from the known configuration of the α-d-mannopyranoside ring, and the absolute structure (Flack) parameter, although not definitive, due to the weak anomalous scattering contributions with MoKα radiation, is consistent with the assignment. Hydrogen atoms attached to O and N atoms were located in a difference Fourier map and refined with isotropic temperature factors. The positions of H atoms attached to C atoms were calculated using idealized *sp*^3^ geometry and included as riding atoms in the least-squares refinement. For the methyl hydrogens, the torsion angle about the C-Me bond was optimized during the refinement.

Solutions in organic solvents were dried with anhydrous Na_2_SO_4_, and concentrated at 40 °C/2 kPa. Combustion analysis was performed by Atlantic Microlab, Inc., Norcross, GA, USA.

*5-(Methoxycarbonyl)pentyl-3-O-benzyl-4-(2,4-di-O-acetyl-3-deoxy-l-glycero-tetronamido)-4,6-dideoxy-2-O-levulinoyl-α-d-mannopyranoside* (**2**). A mixture of Compound **1** (5.7 g, 8.36 mmol) [8], methyl 6-hydroxyhexanoate [9] (1.34 g, 9.19 mmol) and 4 Å MS (1.0 g) in CH_2_Cl_2_ (anhydrous) was stirred at room temperature under Ar for 30 min. TMSOTf (83 μL, 0.46 mmol) was added, and the mixture was stirred for another 5 h. TLC (*R_f_* = 0.3, EtOAc–hexane 1:1) showed that all of **1** was consumed and that a slightly more polar product was formed. Et_3_N (0.5 mL) was added, and the mixture was filtered through a Celite pad. The filtrate was concentrated, and chromatography gave Compound **2** (4.7 g, 84%). The analytical data (^1^H-, ^13^C-NMR, ESI-MS) of **2** agreed with those reported [10].

*5-(Methoxycarbonyl)pentyl-4-(3-deoxy-l-glycero-tetronamido)-4,6-dideoxy-α-d-mannopyranoside* (**3**): Compound **2** (4.5 g, 6.67 mmol) was dissolved in EtOAc–MeOH 1:1 (200 mL), and 5% Pd/C (2.4 g, Escat 103) was added. The mixture was stirred overnight under hydrogen, when TLC showed that the reaction was complete. A slower moving product was formed (*R_f_* = 0.6, 1:1 acetone-hexane). After filtration through a Celite pad and concentration of the filtrate, the syrupy product of debenzylation was dissolved in dry MeOH, and 1 N NaOMe/MeOH was slowly added until pH 10 (~1 mL). The clear solution was kept at room temperature for 1.5 h, when TLC showed that the reaction was complete (*R_f_* = 0.15, 12:1 CH_2_Cl_2_-MeOH). Amberlite IR-120 (H^+^) resin was added in small portions with stirring until pH 7 was reached. After filtration, the filtrate was concentrated, and flash chromatography (40 g silica gel column, 13:1→10:1 CH_2_Cl_2_–MeOH) afforded **3** as a white solid (2.45 g, 93%). Slow crystallization from MeOH gave crystals, which, when dried at 65 °C for 2 h, showed m.p. 128.5–129.5 °C; [α]_D_ +26.3 (*c* 1.1, H_2_O).

^1^H-NMR (600 MHz, D_2_O): δ 4.73 (d, 1H, *J* = 1.2 Hz, H-1), 4.19 (dd, 1H, *J* = 3.6 Hz, 9.0 Hz, H-2'), 3.84–3.82 (m, 2H, H-2, H-3), 3.77–3.75 (m, 2H, H-4, H-5), 3.64 (dd, 2H, *J* = 5.4 Hz, 7.2 Hz, H-4'), 3.61–3.58 (m, 1H, H-1''a), 3.59 (s, 3H, 6''-OMe), 3.43 (dt, *J* = 6.2 Hz, 9.9 Hz, 1H, H-1''b), 2.31 (t, 2H, *J* = 7.8 Hz, H-5''), 1.94–1.91 (m, 1H, H-3'a), 1/78–1.73 (m, 1H, H-3'b), 1.56–1.49 (m, 4H, H-2'', H-4''), 1.31–1.25 (m, 2H, H-3''), 1.08 (d, 2H, *J* = 6.0 Hz, H-6).

^13^C-NMR (150 MHz, D_2_O): δ 177.3 (C-6''), 177.0 (C-1'), 99.5 (C-1), 69.1 (C-2), 68.7(C-2'), 67.7 (C-3), 67.5 (C-1''), 67.1 (C-5), 57.6 (C-4'), 52.6 (C-4), 51.8 (6''-OMe), 35.7 (C-3'), 33.3 (C-5''), 27.8 (C-2''), 24.6 (C-3''), 23.7 (C-4''), 16.5 (C-6).

TOF-HRMS *m/z*: [M+H]^+^ calcd. for C_17_H_32_NO_9_, 394.2072; found, 394.2072.

Anal. calcd. for C_17_H_31_NO_9_: C, 51.90; H, 7.94; N, 3.56. Found: C, 52.07; H, 7.86; N, 3.63.
